# A risk calculator to predict adult attention-deficit/hyperactivity disorder: generation and external validation in three birth cohorts and one clinical sample

**DOI:** 10.1017/S2045796019000283

**Published:** 2019-05-15

**Authors:** A. Caye, J. Agnew-Blais, L. Arseneault, H. Gonçalves, C. Kieling, K. Langley, A. M. B. Menezes, T. E. Moffitt, I. C. Passos, T. B. Rocha, M. H. Sibley, J. M. Swanson, A. Thapar, F. Wehrmeister, L. A. Rohde

**Affiliations:** 1 Department of Psychiatry, Hospital de Clínicas de Porto Alegre, Federal University of Rio Grande do Sul, Brazil; 2 MRC Social, Genetic and Developmental Psychiatry Centre, Institute of Psychiatry, Psychology and Neuroscience, King's College London, UK; 3 Post-Graduate Program in Epidemiology, Federal University of Pelotas, Pelotas, Brazil; 4 Division of Psychological Medicine and Clinical Neurosciences; MRC Centre for Neuropsychiatric Genetics and Genomics, Cardiff University, Cardiff, UK; 5 School of Psychology, Cardiff University, Cardiff, UK; 6 Department of Psychology and Neuroscience, Duke University, Durham, North Carolina, USA; 7 Graduation Program in Psychiatry and Laboratory of Molecular Psychiatry, Federal University of Rio Grande do Sul, Porto Alegre, Brazil; 8 Department of Psychiatry and Behavioral Health at the Florida International University, Herbert Wertheim College of Medicine, US; 9 Department of Pediatrics, University of California, Irvine, USA; 10 National Institute of Developmental Psychiatry for Children and Adolescents, São Paulo, Brazil

**Keywords:** Attention-deficit hyperactivity disorder, child psychiatry, epidemiology, risk factors, statistics

## Abstract

**Aim:**

Few personalised medicine investigations have been conducted for mental health. We aimed to generate and validate a risk tool that predicts adult attention-deficit/hyperactivity disorder (ADHD).

**Methods:**

Using logistic regression models, we generated a risk tool in a representative population cohort (ALSPAC – UK, 5113 participants, followed from birth to age 17) using childhood clinical and sociodemographic data with internal validation. Predictors included sex, socioeconomic status, single-parent family, ADHD symptoms, comorbid disruptive disorders, childhood maltreatment, ADHD symptoms, depressive symptoms, mother's depression and intelligence quotient. The outcome was defined as a categorical diagnosis of ADHD in young adulthood without requiring age at onset criteria. We also tested Machine Learning approaches for developing the risk models: Random Forest, Stochastic Gradient Boosting and Artificial Neural Network. The risk tool was externally validated in the E-Risk cohort (UK, 2040 participants, birth to age 18), the 1993 Pelotas Birth Cohort (Brazil, 3911 participants, birth to age 18) and the MTA clinical sample (USA, 476 children with ADHD and 241 controls followed for 16 years from a minimum of 8 and a maximum of 26 years old).

**Results:**

The overall prevalence of adult ADHD ranged from 8.1 to 12% in the population-based samples, and was 28.6% in the clinical sample. The internal performance of the model in the generating sample was good, with an area under the curve (AUC) for predicting adult ADHD of 0.82 (95% confidence interval (CI) 0.79–0.83). Calibration plots showed good agreement between predicted and observed event frequencies from 0 to 60% probability. In the UK birth cohort test sample, the AUC was 0.75 (95% CI 0.71–0.78). In the Brazilian birth cohort test sample, the AUC was significantly lower –0.57 (95% CI 0.54–0.60). In the clinical trial test sample, the AUC was 0.76 (95% CI 0.73–0.80). The risk model did not predict adult anxiety or major depressive disorder. Machine Learning approaches did not outperform logistic regression models. An open-source and free risk calculator was generated for clinical use and is available online at https://ufrgs.br/prodah/adhd-calculator/.

**Conclusions:**

The risk tool based on childhood characteristics specifically predicts adult ADHD in European and North-American population-based and clinical samples with comparable discrimination to commonly used clinical tools in internal medicine and higher than most previous attempts for mental and neurological disorders. However, its use in middle-income settings requires caution.

## Introduction

Attention-deficit/hyperactivity disorder (ADHD) is consistently associated with an increased risk of several adverse health and social outcomes, including poor education achievement, risky sexual behaviours and premature mortality (Cortese *et al*., 2013; Chang *et al*., 2014; Dalsgaard *et al*., 2015; Faraone *et al*., 2015). ADHD might begin in childhood and persist throughout adulthood, or it may remit spontaneously in around half of the cases (Caye *et al*., 2016*b*). Recent evidence suggested that subthreshold symptoms can get worse over time, causing the emergence of a full-blown syndrome only in adulthood (Caye *et al*., 2017), although the topic is still under debate in the literature (Cooper *et al*., 2018; Manfro *et al*., 2018). Although some risk factors for the persistence or emergence of adult ADHD are known (Caye *et al*., 2016*b*, *c*), the attending psychiatrist is currently unable to correctly predict the course of the disorder based on clinical assessments of children or to propose a preventive intervention for those at risk.

One issue might be the inability to combine what is already known about risk factors. Although mental disorders arise from multiple risk factors, previous studies frequently define risk for targeted preventive interventions on the basis of a single risk factor, for instance, an affected first-degree relative or presence of subthreshold symptoms (Brent *et al*., 2015; Taylor *et al*., 2015; Buntrock *et al*., 2016). Meanwhile, multivariable risk scores such as the Framingham risk score for cardiovascular disease have been one of the main frameworks for the study of preventive strategies in other areas of medicine.

Our aim was to develop and validate a multivariable risk calculator that estimates the individual risk of ADHD in late adolescence/young adulthood based on childhood characteristics. ADHD lends itself easily to the development of a risk calculator for the following reasons: First, its adverse health and social consequences are well established (Asherson *et al*., 2016). Second, it is widely accepted that its roots are in early childhood, although some argue the full syndrome might develop later in some individuals (Moffitt *et al*., 2015; Agnew-Blais *et al*., 2016; Caye *et al*., 2016*a*). Third, being a neurodevelopmental disorder, early intervention has the potential to change brain development and improve later clinical outcomes (Shaw *et al*., 2006). Fourth, there is substantive evidence to support *a priori* hypotheses about specific childhood risk factors (Caye *et al*., 2016*b*).

## Method

Our methods follow well-established probability models in medicine and recommendations of the Transparent Reporting of a multivariable prediction model for Individual Prognosis Or Diagnosis (TRIPOD) statement (Collins *et al*., 2015). We developed the predictive model in one *a priori* selected sample and validated it independently in three external samples (TRIPOD analysis type 3). We selected the Avon Longitudinal Study of Parents and Children (ALSPAC) cohort as the generating sample based on the following *a priori* defined criteria: population-based sample, largest sample.

### Samples and participants

#### ALSPAC

The ALSPAC is a prospective birth cohort study in the UK. Pregnant women with expected delivery dates between 1 April 1991 and 31 December 1992 were invited to participate. Ethical approval for the study was obtained from the ALSPAC Ethics and Law Committee and the Local Research Ethics Committees. Further details on assessments can be found elsewhere (Boyd *et al*., 2013). Please note that the study website contains details of all the data that are available through a fully searchable data dictionary (http://www.bris.ac.uk/alspac/researchers/data-access/data-dictionary/). For the current study, we included 5113 subjects that were assessed for ADHD in childhood (age 7 or 10) and in the last available assessment (age 17).

#### E-Risk

The Environmental Risk (E-Risk) Longitudinal Twin Study is a prospective birth cohort study designed to represent the UK population. In 1999–2000, investigators enrolled 1116 families with same-sex 5-year-old twins (*N*  =  2232) born from 1 January 1994 to 4 December 1995 (Moffitt and Team, 2002). The study was approved by the Joint South London and Maudsley and the Institute of Psychiatry Research Ethics Committee, and parents gave informed written consent. Further details can be found elsewhere (Moffitt and Team, 2002). For the analyses, we included 2040 subjects with data on ADHD in childhood (ages 5, 7, 10 or 12) and in young adulthood (age 18).

#### Pelotas 1993

The 1993 Pelotas Birth Cohort is a prospective longitudinal birth cohort set in Brazil. In 1993, mothers of all children born in the city of Pelotas were contacted and 5249 children were enrolled. The study was approved by the institutional review board of the Federal University of Pelotas, and participants provided written informed consent. Further information on the cohort design can be found elsewhere (Goncalves *et al*., 2014). For the current study, we included 4039 participants that had complete ADHD assessment at age 18–19 years old.

#### MTA

The Multimodal Treatment Study of Children with ADHD (MTA) is the largest clinical trial and observational follow-up conducted with children with ADHD. In the first phase of the study, investigators enrolled 579 children aged 7–10 years old with ADHD and assigned them to 14 months of one of four groups of management. Two years after baseline, 515 consented to enter an observational follow-up and a local normative comparison group of 289 classmates (258 without ADHD) was added. Assessments were conducted at 12, 14 and 16 years after baseline. Informed consent (parental permission and child assent) was obtained for all participating families, using forms approved by both local institutional review boards and the NIH. Detailed design and methods have been presented in previous publications (1999). We included 717 subjects with any complete ADHD assessment in young adulthood (mean age 24).

### Assessment and definition of the outcome variable

In each sample, the outcome was a dichotomous ADHD definition in late adolescence or young adulthood. In ALSPAC, participants' parents completed the hyperactive subscale of the Strengths and Difficulties Questionnaire (SDQ-HS) at 17 years of age. The scale showed excellent discrimination against a DSM-IV diagnosis derived from the Development and Well-Being Assessment (DAWBA) conducted in a subsample of 1673 participants (area under the curve (AUC)  =  0.89, 95% CI 0.81–0.96). The best cut-off score to define diagnosis was at least six points on the SDQ-HS (sensitivity  =  83.3% and specificity  =  83.3%). In the E-Risk, ADHD was ascertained at age 18 years using structured interviews based on full DSM-5 criteria (Agnew-Blais *et al*., 2016). In the MTA sample, ADHD symptoms were derived from the parents’ Conners Adult ADHD Rating Scale (CAARS). At least five DSM-5 symptoms of inattention and/or hyperactivity were required for the symptom criteria. Impairment was evaluated with the Impairment Rating Scale (IRS), which has strong psychometrics and accurately identifies impairment in adults with ADHD (Sibley *et al*., 2012). This diagnostic approach was chosen because it has better diagnostic accuracy than a semi-structured interview in this sample (Sibley *et al*., 2017*b*). In the Pelotas cohort, trained psychologists interviewed the participants at 18–19 years old with a structured interview for ADHD based on DSM-5 criteria (Caye *et al*., 2016*a*). A strict age-at-onset criterion was not required to define ADHD in young adulthood to take into account recent evidence suggesting a significant prevalence of late-onset ADHD presentation (Moffitt *et al*., 2015; Agnew-Blais *et al*., 2016; Caye *et al*., 2016*a*).

### Assessment and definition of predictor variables

We selected the following predictor variables assessed in childhood: female sex, socioeconomic status, mother's depression, intelligence quotient, maltreatment, ADHD symptoms, depressive symptoms, oppositional defiant behaviour and conduct disorders, and single-parent family. All predictors were collected before age 12, with the exception of intelligence in Pelotas, which was measured at age 18. Their selection was based on the extensive review of previous reports in the literature and a meta-analysis conducted by our group (Moffitt *et al*., 2015; Agnew-Blais *et al*., 2016; Caye *et al*., 2016*a*, *b*). We have included all variables that were available across the four samples with some level of comparability, without performing univariate analysis or stepwise techniques for variable selection. Definition of predictors was defined *a priori* according to relevant literature in the field. Further details are provided in online eTable 1.

### Statistical analysis

When developing a predictive model in multiple samples, a recommended approach consists in selecting and tuning the best model in one *a priori* selected sample and assessing its performance in the remaining independent samples for external validity. Because the evaluation of internal performance within the same sample where the model was derived is affected by overfitting, internal validation optimism correction should be performed. Among the most accepted techniques for internal validation is bootstrap resampling.

We have developed the predictive model in the ALSPAC cohort. We ran a logistic regression including outcome (ADHD at last assessment) as the dependent variable and all eligible predictor variables as covariates. We inspected linearity assumptions of continuous variables by plotting the predictor and the logit of the outcome, and trough Box–Tidwell regressions. We derived the model using linear splines of equal sample sizes (with knots at 25^th^, 50^th^ and 75^th^ percentiles) in the ADHD symptom variable, and this model had better fit indices (AIC, BIC). Multiple imputation with chained equations (ten imputations) using the remaining predictors was used to deal with missing values in the predictor variables. We used a fixed number of ten iterations and assessed convergence with trace plots. In the ALSPAC cohort, for each of the 1000 bootstrap resamples, we have performed pooled regression coefficient estimates and variance across imputations with the command *mi estimate* in Stata (Rubin, 1987). We evaluated the predictive discrimination of the probability model calculating the area under the receiver operating characteristic curve (*c*-statistic) of the estimated probability against the actual outcome as an index of model performance. We have assessed optimism of internal validation with bootstrap inference using 1000 replications with the R package *rms* (Harrell *et al*., 1996). We have assessed internal and external model calibration with calibration curves, plotting predicted probabilities against observed frequencies. Extreme predictions at the right end of the distribution (highest risk) including <1% of the sample at risk were excluded of the calibration analyses to avoid instability of the estimates, and these ranges are not shown in each graph. Multiple imputation and model generation were conducted in Stata MP 13.0. Finally, we tested the predictive discrimination of the same predictors using Machine Learning approaches with the R package *caret* (see eMethods).

We performed several sensitivity analyses to assess the robustness of our findings. We analysed the performance (measured by the *c*-statistic) of the model among individuals who endorsed a very low number of ADHD symptoms at baseline (operationalised as equal or below the median of each population) in ALSPAC, E-Risk and Pelotas samples. We had also analysed the performance (measured by the *c*-statistic) of the model excluding one variable at each time. Finally, we present the variation of the predicted probability within fixed levels of ADHD symptoms to assess the contribution of the remaining variables to the model.

## Results

The number of participants with a dichotomous definition of adult ADHD and the frequency of childhood predictors in each sample can be found in Table 1.

**Table 1.** Frequency of young adulthood ADHD and of childhood predictors across the four samples

	ALSPAC (*n* = 5113)	E-Risk (*n* = 2040)	MTA (*n* = 717)	Pelotas (*n* = 4039)
Adult ADHD	486 (9.5%)	166 (8.1%)	205 (28.6%)	492 (12.2%)
Female sex	2619 (51.2%)	1071 (52.5%)	153 (21.3%)	2061 (51.0%)
Socioeconomic status				
Upper	868 (18.6%)	401 (19.7%)	136 (18.9%)	763 (19.6%)
Middle	2172 (46.4%)	966 (47.5%)	356 (50.7%)	1775 (45.6%)
Lower	1637 (35.0%)	665 (32.7%)	210 (29.9%)	1358 (34.9%)
Single parent	519 (11.8%)	450 (22.6%)	190 (26.5%)	882 (22.7%)
ODD or CD	157 (3.4%)	602 (29.5%)	304 (43.6%)	275 (7.0%)
Maltreatment				
Not detected	2084 (41.0%)	1609 (78.9%)	384 (55.3%)	2475 (67.0%)
Probable	2568 (50.5%)	312 (15.3%)	279 (40.1%)	672 (18.3%)
Severe	430 (8.5%)	119 (5.8%)	32 (4.6%)	548 (14.8%)
Lifetime depression of the mother^a^	1850 (36.3%)	990 (48.5%)	326 (48.2%)	1881 (48.4%)
	Mean (SD)	Mean (SD)	Mean (SD)	Mean (SD)
IQ	106.9 (16.3)	98.9 (15.6)	103.1 (19.5)	96.5 (12.5)
	Median (IQR)	Median (IQR)	Median (IQR)	Median (IQR)
Depressive symptoms^b^	0 (1)	1 (2.5)	5.4 (6.7)	4 (4)
Number of ADHD symptoms^c^	2 (6)	1.5 (3.3)	8.3 (9.6)	4 (5)

ADHD, attention-deficit hyperactivity disorder; ODD, oppositional defiant disorder; CD, conduct disorder; SD, standard deviation; IQR, interquartile range; IQ, intelligence quotient.

a Definition of lifetime depression of the mother was designed to be very sensitive, either by multiple assessments and/or by applying a very low threshold (further details on Table S1 of Supplementary material).

b ALSPAC: number of DSM-IV depressive items endorsed. E-Risk, MTA: Children's Depressive Inventory (CDI) score. Pelotas: emotional subscale score of the SDQ.

c ALSPAC, E-Risk, MTA: number of DSM-IV ADHD items endorsed. Pelotas: hyperactivity subscale score of the SDQ.

Note: reported values before multiple imputation. Because each factor may have missing values, we report total number of participants and a proportion where the denominator is the total number of valid subjects.

### Performance of the predictive model in the generating sample

**Fig. 1.**
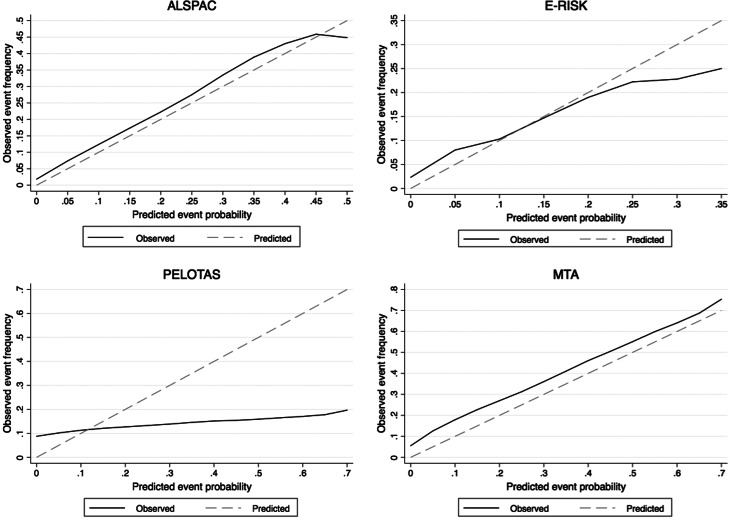
Receiver operating characteristic curves in each each cohort plotting Sensitivity and 1-Specificity for the predicted probabilities generated by the risk calculator against adult ADHD as the classificatory variable.

**Fig. 2.**
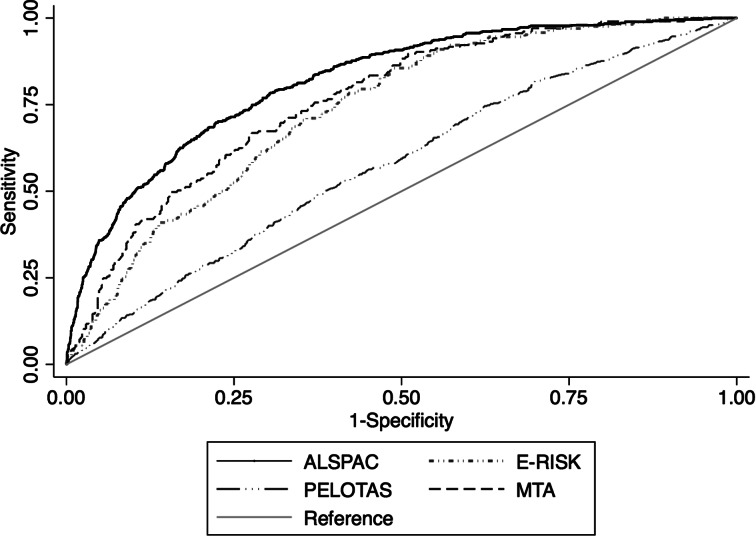
Calibration curves in each cohort plotting the predicted probabilities generated by the risk calculator (x-axis) against observed adult ADHD frequency (y-axis). Dashed diagonal line represents perfect calibration.

All variables entered in the probabific model were used for the calculation of the estimated risk of the individual (Table 2). Only ADHD symptoms were corrected with splines. The predictive model discriminated between adult ADHD *v*. no adult ADHD with an AUC of 0.82 (Bootstrap-corrected 95% CI 0.80–0.83, *p* < 0.001), which indicates very good discrimination (Fig. 1). Correction for optimism with bootstrapping yielded an AUC of 0.81. The calibration plot showed that the predicted probability and the observed frequency of adult ADHD closely agreed throughout the entire range of risk (0 to around 50%, Fig. 2). The bias-corrected calibration curve was nearly identical (eFig. 1). The AUC varied within a range of 0.74–0.82 in sensitivity analyses taking out one predictor at a time (eTable 2 in Supplementary material). Proposed probability cut-offs are presented with sensitivity, specificity, positive predictive value and negative predictive value in eTable 3 in Supplementary material.

**Table 2.** The probability model in the generating sample (*n*  =  5113)

Predictors	OR (BC, 95% CI)	BC *p*-value
Female sex	0.72 (0.58–0.89)	0.003
Socioeconomic status	–	–
Upper social class	*Reference*	–
Middle social class	1.58 (1.15–2.16)	0.004
Lower social class	1.55 (1.11–2.15)	0.010
Single parent family	1.19 (0.90–1.58)	0.215
ADHD symptoms – 0–25^th^	3.77 (2.09–6.79)	<0.001
ADHD symptoms – 25–50^th^	1.19 (1.02–1.40)	0.031
ADHD symptoms – 50–75^th^	1.13 (1.05–1.22)	0.001
ADHD symptoms – 75–100^th^	1.18 (1.12–1.25)	<0.001
ODD or CD	1.81 (1.21–2.71)	0.004
Childhood maltreatment	–	–
No detected maltreatment	*Reference*	–
Probable maltreatment	1.28 (1.01–1.64)	0.045
Severe maltreatment	1.35 (0.93–1.95)	0.115
Depression of the mother	1.41 (1.13–1.75)	0.002
Intelligence quotient^a^	0.89 (0.85–0.95)	<0.001
Depressive symptoms (*z*-score)^b^	1.00 (0.92–1.10)	0.940

OR, odds ratio; ODD, oppositional defiant disorder; CD, conduct disorder; ADHD, attention-deficit hyperactivity disorder; BC, Bootstrap-corrected.

a We report the OR for a ten-point change in the intelligence quotient scale.

b Due to the OR of 1.00 for depressive symptoms, we have omitted this variable from the online calculator.

### Performance of the predictive model in a validating cohort sample in the same country

In the E-Risk study, the predictive model discriminated between adult ADHD *v*. no adult ADHD with an AUC of 0.75 (Bootstrap-corrected 95% CI 0.71–0.78, *p* < 0.001), which indicates fair discrimination (Fig. 1). The calibration plot showed reasonable agreement between predicted and observed event frequencies, especially in the lower range of risk (Fig. 2). The discrimination was the same when restricting the sample to randomly selected non-siblings (eTable 4 in Supplementary material).

### Performance of the predictive model in a validating sample in a middle-income country

In the Pelotas cohort, the predictive model discriminated between adult ADHD vs. no adult ADHD with an AUC of 0.57 (Bootstrap-corrected 95% CI 0.55–0.60, *p* < 0.001), which indicates poor discrimination (Fig. 1). There was a low agreement between estimated probability and observed frequency of the outcome (Fig. 2).

### Performance of the predictive model in a validating clinical sample in a country with similar income

In the MTA, the predictive model discriminated between adult ADHD *v*. no adult ADHD with an AUC of 0.76 (Bootstrap-corrected 95% CI 0.73–0.80, *p* < 0.001) (Fig. 1). The calibration plot showed that predicted probability and observed frequency of adult ADHD closely agreed throughout the entire range of risk (0 to around 70%, Fig. 2), although the model had underestimated event frequency consistently.

### Performance of the predictive model within participants with very low endorsement of ADHD symptoms in childhood

We tested the performance of the model for predicting late-onset ADHD in population samples, among only participants that endorsed few ADHD symptoms in childhood – the median or lower number of symptoms in their respective populations. The model had fair discrimination in these subgroups, except for the Pelotas sample in which the model already had poor discrimination (Table 3).

**Table 3.** Performance of the score for individuals with very low ADHD childhood symptoms

	AUC	BC, 95% CI	BC *p*-value
ALSPAC (*n* = 2688)	0.77	0.72–0.82	<0.001
E-Risk (*n* = 1099)	0.78	0.71–0.86	<0.001
Pelotas (*n* = 2135)	0.56	0.52–0.60	<0.001

BC, Bootstrap-corrected.

ROC analyses were done only in participants with low endorsement of ADHD symptoms in childhood. Low endorsement was defined as a median number of symptoms or below the median of their respective population (ALSPAC: two or less ADHD symptoms; E-Risk: one or zero ADHD symptoms; Pelotas: the median or less than median (4) in the hyperactivity subscale of the SDQ).

### Performance of the predictive model removing one predictor at a time

We tested the model taking out one predictor at a time (eTable 2). The most relevant individual predictor was the level of ADHD symptoms in childhood. However, the model still had fair performance in the model without ADHD symptoms in childhood, with an AUC of 0.74 (95% CI 0.72–0.76, *p* < 0.001).

### Variation of the predicted probability within fixed levels of ADHD symptoms

We assessed the predicted probabilities of an adult ADHD diagnosis at any fixed level of ADHD symptoms, considering a maximum variation of the remaining factors (see eFig. 2). The observed variance indicates that ADHD symptoms are not the only relevant predictive factor in the model. These findings analysed together clearly indicate that this is not a model based on just one variable.

### Specificity of the predictive model in predicting ADHD

Considering that E-risk is the population cohort with the most comprehensive assessment of comorbid mental disorders, we tested the model's discrimination predicting adult anxiety and major depressive disorder. The performance was significantly lower than for ADHD, showing specificity for ADHD compared to other forms of adult psychopathology (eTable 5 in Supplementary material).

### Risk calculator and robustness of findings

Predictive discrimination estimates using three different Machine Learning approaches were almost the same (see eTable 6 in Supplementary material). In a secondary analysis, we also have developed one comprehensive predictive model with all samples at once, using site as one more predictor variable (see eTable 7; eFig. 3). A risk calculator can be found at http://www.ufrgs.br/prodah/adhd-calculator/.

## Discussion

The widespread use of tools that predict clinical outcomes in medical practice has promoted the development and testing of preventive interventions, but this approach has been rarely attempted for mental health (Bitton and Gaziano, 2010). We generated a probability model to predict adult ADHD in a large birth cohort in the UK, with very good discrimination – AUC of 0.81 after optimism correction – and calibration. This performance is compared to the most used clinical tools in medicine (Morrow *et al*., 2000). Recent attempts for mental health reported risk scores with good calibration (Fusar-Poli *et al*., 2017; Hafeman *et al*., 2017). These studies lacked, however, a consistent external validation with completely independent samples.

Our next step was to validate the score in independent samples. First, we tested the score in another UK birth cohort, the E-Risk. Its performance in predicting adult ADHD was similar. This is an important finding because several risk models in mental health did not replicate well even in samples from similar settings (Kivipelto *et al*., 2006; Anstey *et al*., 2014). Since data generated in population samples frequently do not translate to clinical samples (Weissman *et al*., 2011), we tested the performance of the score in the MTA study, the largest clinical trial ever conducted for ADHD. As for ALSPAC and E-risk, the score worked well with good discrimination and calibration.

We then tested the score in a third birth cohort from Brazil. We observed that the score was much less accurate with an AUC of 0.57. This finding is not surprising, since previous evidence suggests that the predictive discrimination of risk tools is lower in diverse sociocultural and ethnic populations (Chia *et al*., 2015). However, since predictor factors assessment in Pelotas was the most heterogeneous, observed low discrimination might have been an effect of measurement error.

Models that predict a diagnosis of chronic disorders often include premorbid signs and symptoms of the disease as predictive factors. For example, the factor that increased discrimination the most in the recently published calculator for psychosis was the index diagnosis when presenting to secondary care, where psychotic disorders had the greatest weight compared to other disorders such as mood disorders (Fusar-Poli *et al*., 2017). Although this is a valid approach, other variables must also add to prediction, otherwise models would be tautological. Therefore, we also validated the score in subjects with a low endorsement of ADHD symptoms in childhood. The performance was good even in this sensitivity analysis. In addition, we assessed the probabilities of an adult ADHD diagnosis at any fixed level of ADHD symptoms, allowing maximum variation of the remaining factors. Finally, we checked discrimination of the model removing each factor at once. Findings suggested that although ADHD symptoms are the most important overall predictor, the complete model works as a necessary refinement and a model without ADHD symptoms has good discrimination as well.

We also conducted other secondary analyses to assess the robustness of our findings. We tested the impact of using other statistical methods on our results. We observed that the discrimination of the prediction models remained stable regardless of chosen statistical methods. Finally, we tested the hypothesis of whether the score was specific for the prediction of ADHD. This is an important proof-of-concept: personalised medicine has always been a challenge for the area of psychiatry, as it has been shown consistently that most identified biomarkers and risk factors associated with one mental disorder are also associated with several others (Cross-Disorder Group of the Psychiatric Genomics C *et al*., 2013). We observed that the score was specific for ADHD, not predicting major depressive disorder or anxiety disorders.

Previous cohort investigations included in the present study did not find a significant childhood DSM dichotomous ADHD diagnosis in the trajectory of late-onset ADHD (Agnew-Blais *et al*., 2016; Caye *et al*., 2016*a*). Thus, it might seem surprising that childhood ADHD symptoms predict adult ADHD. The MTA report also highlighted the importance of child ADHD subthreshold symptoms in adult ADHD in cases where formal DSM diagnosis was not found in childhood (Sibley *et al*., 2017*a*). Since this approach was not the main focus of previous cohort studies (ADHD subthreshold symptoms), this might explain why childhood ADHD symptoms predict adult ADHD even in cohorts where childhood dichotomous diagnosis was not relevant for adult ADHD.

Our findings should be interpreted considering a set of limitations. First, the design and assessments of different samples were not uniform, limiting the discrimination of the score in the validating samples. Adult ADHD, for instance, was measured with a scale rather than with a structured interview in the generating sample, but not in the validating samples. It is possible, therefore, that the proposed estimated predictive discrimination in validating samples might actually be an underestimation. Further validating efforts with assessments that more closely resemble those of the generating sample might observe higher AUCs. However, this could also be seen as the strength of the study, since observed discrimination indices are considered good, even with different methodologies implemented in individual studies. Second, there was attrition in the generating sample's assessments. Nevertheless, potential selection bias does not appear to affect the prediction of outcomes in this cohort, as shown in previous publications (Boyd *et al*., 2013). Also, we have used multiple imputation techniques to deal with missing values. Third, the observed positive predictive value in selected cut-offs reaches a maximum of 61.8%, while the negative predictive value is much higher throughout prediction. Although this might be considered insufficient, we ought to remember that the positive predictive value depends much on the prevalence of the studied condition, and we are working with population-based samples where the base rate of the condition is low. As a comparison, the Framingham risk score, which is also a tool developed in the general population, yields a positive predictive value of up to 30–40%. The risk score for bipolar disorder reports a positive predictive value of up to 32%, even among offspring of bipolar patients (a high-risk sample). Fifth, it is important to note that other variables that are related to ADHD could have been a part of the risk score like prematurity and ADHD in first-degree relatives. However, they were not available for testing in the four data sets and our guide for risk factors was evidence-based guided by a previous meta-analysis (Caye *et al*., 2016*b*). Accordingly, the predicted probability provided by the model should be considered an estimate probability obtained with a pre-specified set of variables.

What is the clinical utility of this score, provided that previous literature already has shown that most variables included in our model that are non-specific risk factors for mental disorders and ADHD symptoms in childhood, as expected, are key predicted risk factors for adult ADHD? No the previous effort combined all these variables in a single risk calculator. Therefore, the only information that clinicians could offer was that some variables, like comorbidity with CD/ODD in childhood, increase the risk of persistence of ADHD. By using this calculator, attending clinicians can identify high-risk individuals to inform parents and guide decisions.

Thus, we propose a multivariable risk model to predict ADHD in young adulthood based on childhood factors that have good discrimination in both population and clinical settings. Clinicians can use the model to guide long-term decisions based on the identification of children at high risk for future adult ADHD diagnosis. Also, it provides a framework for testing the effectiveness of preventive interventions focused on high-risk individuals. Furthermore, the score might be used to identify at-risk individuals for investigating neurobiological features including brain development. The lower discrimination observed in a middle-income country urges the discussion of how globally generalisable are the risk models that are currently being widely used in clinical practice. Indeed, even the well-established Framingham cardiovascular risk model is being subjected to criticism for its wide variation in performance across different populations. Therefore, future attempts to improve the current model should include setting-specific recalibration analyses that should then be translated to specific risk calculators to be used across different settings. Also, we suggest that cohorts use more standardised methods of collection of predictors and outcomes in psychiatry for the study of risk factors, so that we can disentangle whether failure to replicate is due to the heterogeneity of methods or population. Hence, our work adds to the need for validation of risk models in low- and middle-income countries.

## Supplementary material

For supplementary material accompanying this paper visit http://dx.doi.org/10.1017/S2045796019000283. click here to view supplementary material

## Availability of data and material

Due to constraints on the data sharing permissions of the samples included in this study, we are not allowed to share the data for public use.
