# A risk calculator to predict adult attention-deficit/hyperactivity disorder: generation and external validation in three birth cohorts and one clinical sample - ERRATUM

**DOI:** 10.1017/S2045796019000337

**Published:** 2019-07-03

**Authors:** A. Caye, J. Agnew-Blais, L. Arseneault, H. Gonçalves, C. Kieling, K. Langley, A. M. B. Menezes, T. E. Moffitt, I. C. Passos, T. B. Rocha, M. H. Sibley, J. M. Swanson, A. Thapar, F. Wehrmeister, L. A. Rohde

**Affiliations:** 1 Department of Psychiatry, Hospital de Clínicas de Porto Alegre, Federal University of Rio Grande do Sul, Brazil; 2 MRC Social, Genetic and Developmental Psychiatry Centre, Institute of Psychiatry, Psychology and Neuroscience, King's College London, UK; 3 Post-Graduate Program in Epidemiology, Federal University of Pelotas, Pelotas, Brazil; 4 Division of Psychological Medicine and Clinical Neurosciences; MRC Centre for Neuropsychiatric Genetics and Genomics, Cardiff University, Cardiff, UK; 5 School of Psychology, Cardiff University, Cardiff, UK; 6 Department of Psychology and Neuroscience, Duke University, Durham, North Carolina, USA; 7 Graduation Program in Psychiatry and Laboratory of Molecular Psychiatry, Federal University of Rio Grande do Sul, Porto Alegre, Brazil; 8 Department of Psychiatry and Behavioral Health at the Florida International University, Herbert Wertheim College of Medicine, US; 9 Department of Pediatrics, University of California, Irvine, USA; 10 National Institute of Developmental Psychiatry for Children and Adolescents, São Paulo, Brazil

In the aforementioned article, the figures have been incorrectly inverted. The correct figures and their corresponding captions are as follows:

**Fig. 1.**
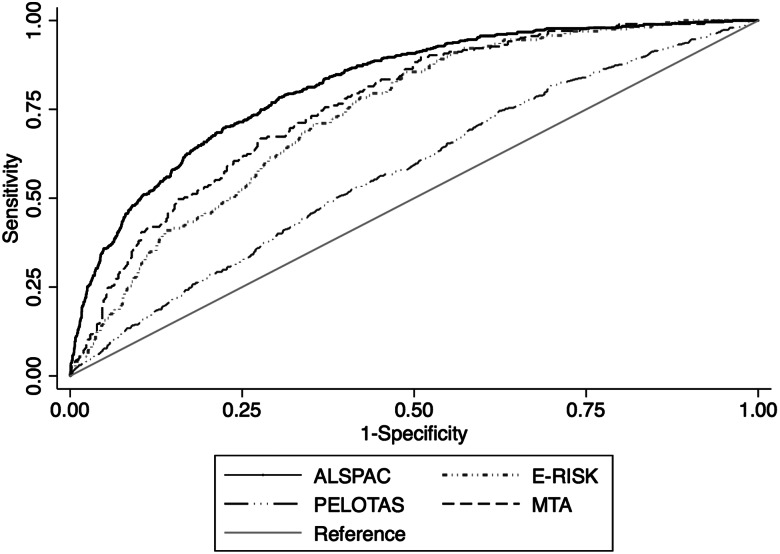
Receiver operating characteristic curves in each each cohort plotting Sensitivity and 1-Specificity for the predicted probabilities generated by the risk calculator against adult ADHD as the classificatory variable.

**Fig. 2.**
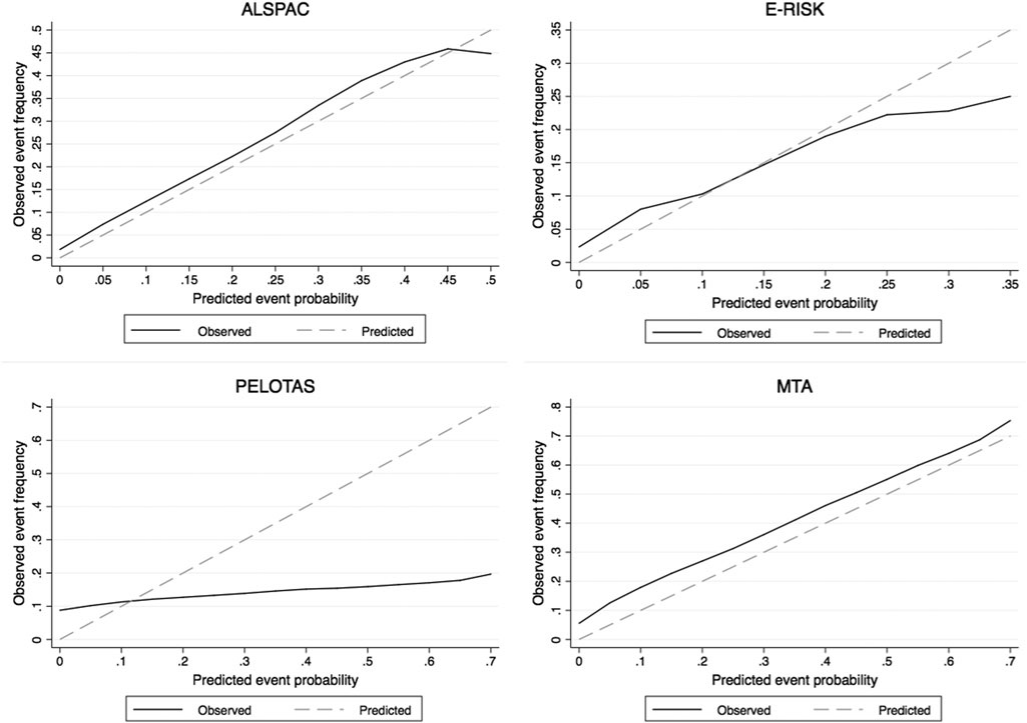
Calibration curves in each cohort plotting the predicted probabilities generated by the risk calculator (x-axis) against observed adult ADHD frequency (y-axis). Dashed diagonal line represents perfect calibration.

The publisher regrets this error.
