# Supplementary material for: A risk calculator to predict adult attention-deficit/hyperactivity disorder: generation and external validation in three birth cohorts and one clinical sample
Source: Epidemiol Psychiatr Sci. 2019 May 15;29:e37. doi: 10.1017/S2045796019000283 (PMC8061253; doi:10.1017/S2045796019000283)
Supplement: Supplementary file 1 [file S2045796019000283sup001.docx]

**Supplemental material of “A risk calculator to predict adult Attention-deficit/Hyperactivity disorder: generation and external validation in three birth cohorts and one clinical sample”.**

Contents:

| 1. eTable 1. Assessment of predictor variables. |  |
| --- | --- |
| 1. eMethods: Machine learning approaches: methods |  |
| 1. eFigure 1. Bias-corrected calibration plot for internal validation in the ALSPAC cohort. |  |
| 1. eTable 2. Predictive accuracy of the score leaving out predictors one at a time. |  |
| 1. eTable 3. Sensitivity, specificity, positive and negative predictive values at selected risk cut-offs. |  |
| 1. eTable 4. Assessment of the confounding effect of twin pairs in the E-Risk. |  |
| 1. eFigure 2. Variation of predicted probabilities within fixed levels of ADHD symptoms |  |
| 1. eTable 5. Performance of the score for Major Depression Disorder or Anxiety Disorders in young adulthood. |  |
| 1. eTable 6 Performance of the predictive model using Machine Learning approaches. |  |
| 1. eTable 7. A comprehensive predictive model |  |
| 1. eFigure 3. Bias-corrected calibration plot for internal validation in the comprehensive model. |  |

Arthur Caye, MD; Jessica Agnew-Blais, PhD; Louise Arseneault, PhD; Helen Gonçalves, PhD; Christian Kieling, MD PhD; Kate Langley, PhD; Ana M. B. Menezes, PhD; Terrie E. Moffitt, PhD; Ives Cavalcante-Passos, MD PhD; Thiago Botter-Maio Rocha, MD MSc; Margaret Sibley, PhD; James M. Swanson, MD, PhD; Anita Thapar, MD PhD; Fernando Wehrmeister, PhD; Luis Augusto Rohde, MD PhD

**eTable 1. Assessment of predictor variables.**

|  | **ALSPAC** | **E-Risk** | **MTA** | **Pelotas** |
| --- | --- | --- | --- | --- |
| Intelligence quotient | Weschler Intelligence Scale for Children version III, age 8 | Weschler Intelligence Scale for Children III-R, Age 12 | Weschler Intelligence Scale for Children version III, ages 7 to 10 | Weschler Adult Intelligence Scale, age 18 |
| ODD or CD | DSM-IV criteria, age 10 | DSM-IV criteria, ages 5 to 12 (or rule) | DSM-IV criteria, ages 7 to 12 (or rule) | SDQ-C >= 7, age 11.^a^ |
| Depressive symptoms | DSM-IV (DAWBA), z-score of symptoms, age 10 | CDI, z-score, age 12 | CDI, z-score of mean reported symptoms, ages 7 to 12^a^ | SDQ-E rated by parents, z-score, age 11 |
| ADHD symptoms | DSM-IV (DAWBA) rated by parents, number of symptoms, age 10 | DSM-IV rated by parents, z-score of number of reported symptoms, age 12 | DSM-IV (DISC-IV), ages 7 to 12, z-score of mean reported symptoms^a^, ages 7 to 12 | SDQ-H rated by parents, z-score, age 11 |
| Childhood maltreatment | Physical, emotional or sexual abuse and maladaptive parenting according to previous definitions (Lereya *et al.*, 2015). None, probable or severe if neither, one or both were present (ages 18 months to 7 years) | None, probable or severe according to previous definitions (Caspi *et al.*, 2003), children’s ages 5 to 12. | Parent-Child Relationship Scale answered by parents ages 7 to 12, grouped into none, probable or severe. | None, probable or severe according to previous definitions (Caspi *et al.*, 2003). Asked retrospectively at age 15. |
| Depression of the mother | Positive if any of the following true: self-reported having had severe depression (age 11); self-reported having taken pills for depression in the last three years (age 9); EDPS of at least 10 (ages 8 months, 18 months). | DSM-IV, children’s ages 5 to 12. | Positive if biological mom retrospectively reported having the blues at or after delivery, asked at baseline (children’s age 7 to 10). | At least 7 points in the SRQ-20, as previously suggested (WHO, 1994), age 11. |
| Social class^c^ | Registrar’s General Classification, asked at birth. | Acorn classification, ages 5 to 12. | Gross household income in US$, age 7 to 10, ages 7 to 10. | ABEP criteria, age 11 |

CDI *Children’s Depression Inventory* | SDQ-C *Strengths and Difficulties Questionnaire, conduct subscale* | SDQ-E *Strengths and Difficulties Questionnaire, emotional subscale* | SDQ-H *Strengths and Difficulties Questionnaire, hyperactivity subscale* | EDPS - *Edinburgh Postnatal Depression Scale* | SRQ-20 *Self-reporting Questionnaire 20* | ABEP *Associação Brasileira de Empresas de Pesquisa (the Brazilian Association of Research Companies)*

1. We ran a ROC curve analyses of the SDQ-C rated by parents against DAWBA in a subsample of children (n = 290). Discrimination was fair (0.77). The best selected cut-off was at least 7 points.
2. Since categories of risk were heterogeneous across studies, we have decided to group categories aiming to achieve similar percentages of the population included in each group. Therefore, the observed effect reflects rather the *relative* social class (how the individual family compares to the population) than the *absolute* concept (how much does the family actually earn or possess).

**eMethods: Machine Learning approaches**

We compared the logistic regression analysis to some well-established machine learning algorithms in order to assess the consistency of our findings. We used the package caret (Version 6.0-73) from R software (<https://www.R-project.org/>). We selected the caret package due to its automated tuning methods for machine learning algorithms, which enable the selection of the best fit for each model. We assumed a diagnostic classification problem with the abovementioned predictors used as input data. The main objective was to train a set of machine learning algorithms to estimate the probability of a subject belonging to either ADHD or healthy control groups given previously unseen subjects’ data. In the present analysis, besides logistic regression, Random Forest, Artificial Neural Network, and Stochastic Gradient Boosting were used because 1) they are capable of modeling more complex patterns than nearly any algorithm; 2) they can handle categorical or continuous features; 3) they can be used on data with extremely large number of observations; 4) they can be used to classification prediction problems. Fforest (or decision tree forests) is an ensemble-based method that focuses only on ensembles of decision trees. This method was developed by Leo Breiman and Adele Cutler, and combines the base principles of “bagging” with random feature selection to add additional diversity to the decision tree models. An Artificial Neural Network (ANN) models the relationship between a set of input signals and an output signal using a model derived from our understanding of how a biological brain responds to stimuli from sensory inputs. Stochastic gradient boosting is another “bagging” procedure.^5^Machine learning approach was conducted in two phases: 1) training and validation phase and 2) test phase. In the first phase, we used the ALSPAC dataset to train, to validate, and to identify the best fit (parameter tuning) for each model. The parameters to be adjusted were 1) size and decay for ANN, 2) mtry (an optional integer specifying the number of features to randomly select at each split) for random forest, and 3) n.trees, interactions.depth, shrinkage, and n.minobsinnode for Stochastic Gradient Boosting. We used optimism bootstrapping (n=1000) as the resampling method and AUC to select the best fit for each model. In the second phase, we tested the selected models in E-Risk, MTA, and Pelotas datasets.

The parameters selected during the first phase were 1) mtry=3 for random forest, 2) size=1 and decay=0.01 for ANN, 3) n.trees=150, interactions.depth=1, shirinkage=0.1, n.minobsinnode=10 for Stochastic Gradient Boosting. eTable6 shows the AUC for each model in all test datasets.

**eFigure 1. Bias-corrected calibration plot for internal validation in the ALSPAC cohort.**

**
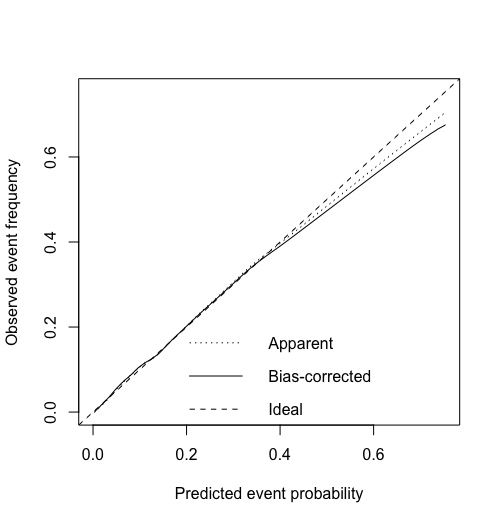
**

**eTable 2. Predictive discrimination of the score leaving out predictors one at a time.**

|  | **AUC** | **95% CI** | **p-value** |
| --- | --- | --- | --- |
| *Full model* | ·82 | ·79 - ·84 | < ·001 |
| - ADHD symptoms | ·74 | ·72 - ·76 | < ·001 |
| - Gender | ·81 | ·80 - ·83 | < ·001 |
| - Social class | ·82 | ·80 - ·83 | < ·001 |
| - Single parent | ·82 | ·80 - ·83 | < ·001 |
| - ODD/CD | ·81 | ·80 - ·83 | < . ·001 |
| - Childhood maltreatment | ·82 | ·80 - ·83 | < ·001 |
| - Depressive symptoms | ·82 | ·79 - ·83 | < ·001 |
| - Mother’s depression | ·81 | ·79 - ·84 | < ·001 |
| - IQ | ·81 | ·79 - ·83 | < ·001 |

AUC *Area under the Curve* | CI *Confidence Interval* | ADHD *Attention-deficit/Hyperactivity Disorder* | ODD *Oppositional Defiant Disorder |* CD *Conduct Disorder*

**eTable 3. Sensitivity, specificity, positive and negative predictive values at selected risk cut-offs.**

|  | **Sensitivity** | **Specificity** | **PPV** | **NPV** |
| --- | --- | --- | --- | --- |
| Probability >= 10% | 72·4% | 74·3% | 22·9% | 96·3% |
| Probability >= 20% | 45·1% | 91·0% | 34·4% | 94·0% |
| Probability >= 30% | 30·3% | 96·0% | 44·5% | 92·9% |
| Probability >= 40% | 20·4% | 98·1% | 52·4% | 92·1% |
| Probability >= 50% | 11·1% | 99·0% | 54·5% | 91·4% |
| Probability >= 60% | 6·0% | 99·6% | 61·8% | 91·0% |

PPV+ *Positive Predictive Value* NPV *Negative Predictive Value*

**eTable 4. The assessment of the confounding effect of twin pairs in the E-Risk.**

|  | **AUC** | **95% CI** | **p-value** |
| --- | --- | --- | --- |
| Random non-siblings 1 (n = 1020) | ·75 | ·70 - ·80 | < 0·001 |
| Random non-siblings 2 (n = 1020) | ·75 | ·70 - ·80 | < 0·001 |

AUC *Area under the Curve* | CI *Confidence Interval* Note: We tested the risk score in the E-Risk sample in subgroups of randomly selected non-sibling participants.

**eFigure 2: Variation of predicted probabilities within fixed levels of ADHD symptoms**

**eTable 5. Performance of the score for Major Depression Disorder or Anxiety Disorders in young adulthood¹.**

|  | **AUC** | **95% CI** | **p-value** | **vs. ADHD (p-value)²** |
| --- | --- | --- | --- | --- |
| Anxiety Disorders | ·52 | ·47 - ·59 | ·72 | < 0·001 |
| Major Depressive Disorder | ·56 | ·52 - ·59 | ·001 | < 0·001 |
| Alcohol Use Disorder | .58 | .54 - .62 | < .001 | < .001 |
| Marijuana Use Disorder | .67 | .60 - .73 | < .001 | .03 |

AUC *Area under the Curve* | CI *Confidence Interval* | ADHD *Attention-deficit/Hyperactivity Disorder*

1. Tested in the E-Risk sample.
2. Tested against the performance of the score for predicting ADHD in the E-Risk cohort

**eTable 6. Performance of the predictive model using Machine Learning approaches.**

|  | **Area Under the Curve (95% Confidence Interval)** | | | |
| --- | --- | --- | --- | --- |
|  | **ALSPAC** | **E-Risk** | **MTA** | **Pelotas** |
| Logistic Regression (original) | ·82 (·80 - ·83) | ·75 (·71 - ·78) | ·76 (·73 - ·80) | ·57 (·54 - ·60) |
| Random Forest | ·80 (·74 - ·87) | ·70 (·67 - ·74) | ·72 (·68 – ·76) | ·56 (·53 - ·59) |
| Stochastic Gradient Boosting | ·81 (·78 - ·83) | ·74 (·71 – ·77) | ·76 (·72 - ·79) | ·57 (·55 – ·60) |
| Artificial Neural Network | ·81 (·77 - ·85) | ·74 (·70 - ·77) | ·76 (·72 - ·80) | ·58 (·55 - ·61) |

**eTable 7. A comprehensive model including all samples**

We have developed an alternative approach using all data at once, including site as a tenth predictor.

| **Predictor** | **OR (BC 95% CI)** | **BC p-value** |
| --- | --- | --- |
| Female sex | 1·06 (·94 – 1·21) | ·349 |
| Social class | - | - |
| Higher | *reference* |  |
| Middle | 1·04 (·87– 1·24) | ·639 |
| Lower | 1·02 (·84 – 1·23) | ·850 |
| Single parent family | 1·09 (·94 – 1·26) | ·262 |
| Childhood maltreatment | - | - |
| None | *reference* | - |
| Probable | 1·39 (1·21 – 1·59) | < ·001 |
| Severe | 1·98 (1·65 – 2·37) | < ·001 |
| Oppositional Defiant Disorder or Conduct Disorder | 1·14 (·97 – 1·34) | ·104 |
| Mother’s depression | 1·12 (·99 – 1·28) | ·083 |
| ADHD symptoms – 0-25^th^ | 2·19 (1·55 – 3·11) | < ·001 |
| ADHD symptoms – 25-50^th^ | 1·21 (1·09 – 1·34) | < .001 |
| ADHD symptoms – 50-75^th^ | 1·11 (1·06 – 1.16) | < .001 |
| ADHD symptoms – 75-100^th^ | 1.07 (1.04 – 1·10) | < ·001 |
| Intelligence quotient^a^ | ·91 (·88 – 1·10) | < ·001 |
| Depressive symptoms | 1·04 (·98 – 1·10) | ·180 |
| Site |  |  |
| ALSPAC | *reference* | - |
| E-Risk | ·99 (·80 – 1·22) | ·944 |
| Pelotas | ·71 (·59 - ·85) | < ·001 |
| MTA | 2·35 (1·89 – 2·92) | < ·001 |

*BC* Bootstrap corrected

1. We report the OR for a 10-point change in the intelligence quotient scale.

Overall Area Under the Curve: ·74 (·73 - ·76), p < ·001 (Bootstrap optimism-corrected: ·73)

We also ran a comprehensive model including all two-way interactions between site and predictor variables, with an AUC of ·78 (available upon request).

**eFigure 3. Bias-corrected calibration plot for internal validation in the comprehensive model.**

**
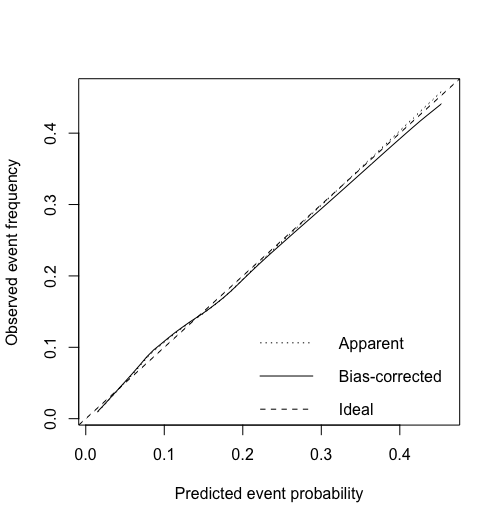
**

**Caspi, A., Sugden, K., Moffitt, T. E., Taylor, A., Craig, I. W., Harrington, H., McClay, J., Mill, J., Martin, J., Braithwaite, A. & Poulton, R.** (2003). Influence of life stress on depression: moderation by a polymorphism in the 5-HTT gene. *Science* **301**, 386-9.

**Lereya, S. T., Copeland, W. E., Costello, E. J. & Wolke, D.** (2015). Adult mental health consequences of peer bullying and maltreatment in childhood: two cohorts in two countries. *Lancet Psychiatry* **2**, 524-31.

**WHO** (1994). A user’s guide to self-reporting questionnaires. . WHO: Division of mental health: Geneva.
